# Unruptured myelomeningocele: a rare clinical image

**DOI:** 10.11604/pamj.2022.43.171.38082

**Published:** 2022-12-05

**Authors:** Lalmalsawmi Khiangte, Pratibha Wankhede

**Affiliations:** 1 Department of Community Health Nursing, Smt Radhikabai Meghe Memorial College of Nursing, Datta Meghe Institute of Medical Sciences, Sawangi (Meghe), Wardha, Maharashtra, India

**Keywords:** Neurosurgery, diagnosis, neural tube, myelomeningocele, sac

## Image in medicine

Incomplete closure of the spinal neural tube during the first month of pregnancy usually causes myelomeningocele to develop during embryonic development. It eventually results in exposed meninges or neural tissue at the level of the damaged vertebra, along with a fluid-filled sac that protrudes. Because the spinal cord does not close, many of the axons of nerves are exposed, resulting in damage to the cord as the pregnancy continues. The prognosis is frequently worse if detected late or is not treated because it can cause terrible morbidity and several impairments such as traumatic birth. An 8-year-old male child was brought to the outpatient department with complaint of swelling on the lower back which is present since birth and is increasing, which was about 15x10 cm. Physical examination was performed by the physician and the finding shows that swelling was soft with dimple and no discharge. Hence, after physical examination, the patient was diagnosed as unruptured myelomeningocele, and was referred to pediatric department for further management.

**Figure 1:**
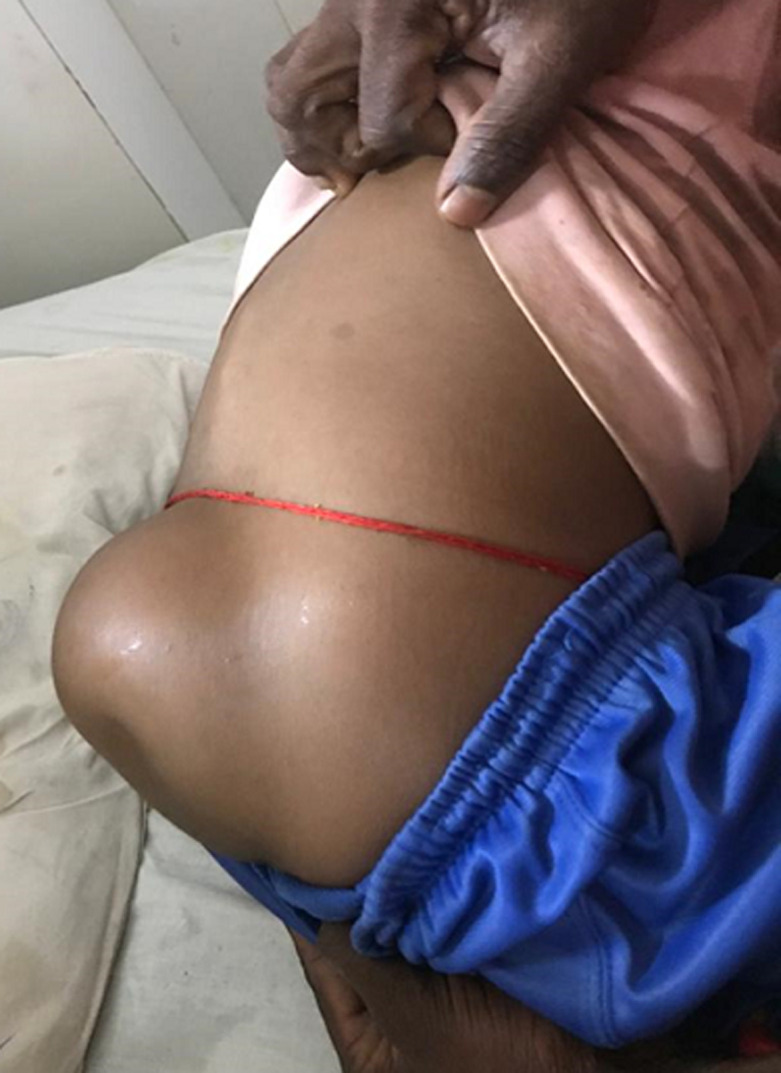
swelling over the lower back

